# Editorial: The impact of non-pharmaceutical interventions (NPIs) on communicable diseases

**DOI:** 10.3389/fpubh.2023.1270709

**Published:** 2023-08-24

**Authors:** Salah T. Al Awaidy, Ramy Mohamed Ghazy, Ozayr Mahomed, Ronald Wesonga

**Affiliations:** ^1^Office of Health Affairs, Ministry of Health, Muscat, Oman; ^2^Tropical Health Department, High Institute of Public Health, Alexandria University, Alexandria, Egypt; ^3^Department of Epidemiology and Population Health, Diabetes Institute, Kuwait City, Kuwait; ^4^Department of Public Health Medicine, University of KwaZulu Natal, Durban, South Africa; ^5^Department of Statistics, College of Sciences, Sultan Qaboos University, Muscat, Oman

**Keywords:** non-pharmaceutical interventions, communicable diseases, COVID-19, pandemic, outbreaks

Non-pharmaceutical Interventions (NPIs) are strategies that individuals and communities use to reduce the spread of diseases, such as outbreaks or pandemics. The purpose of this collection was to develop a reference for the most recent high-quality publications in the field of NPIs through assessing the effectiveness, challenges, and lessons gleaned from NPI implementations. The themes encompassed comprehensive examination of NPIs efficacy during COVID-19, strategies to bolster the evidence supporting their effectiveness, the psychosocial ramifications of prolonged NPIs, the influence on healthcare accessibility, NPIs' potential to mitigate future outbreaks, and their role in reducing morbidity and mortality in countries. Additionally, the research collection explored the utilization and public perception of NPIs, such as mask-wearing and hand hygiene, as well as the impact of being homebound and telecommuting during pandemics.

Zhunis et al. introduced a novel approach using probabilistic programming to assess the effectiveness of major initial NPIs. Their generative simulation model considered both the economic and human capital costs associated with implementing these strategies. They created a pipeline to simulate virus spread and the resulting losses under various policy combinations. The study identified social distancing combined with contact tracing as the most successful policy, leading to a remarkable 96% reduction in virus transmission rate and a 98% decrease in economic and human capital losses. To support their findings, the researchers made their framework available as open source, allowing others to test the efficacy of different policy combinations.

The International Citizen Project on COVID-19 consortium conducted online surveys in six sub-Saharan African countries, between March and August 2020. They assessed individual adherence to measures like mask-wearing, physical distancing, hand hygiene, coughing etiquette, and avoiding face touching, as well as community behaviors such as going to public places and traveling during the pandemic. The data from a sample of 26,678 respondents were analyzed. The results showed that the mean individual adherence score declined from 3.80 ± 1.37 during Period 1 (March–May) to 3.57 ± 1.43 during Period 2 (June–August), indicating reduced adherence over time. At the community level, there was a significant increase in attendance at public events and more travel during period 2 compared to period 1. Using linear mixed models, the study found that higher age, female gender, higher educational level, and working in the healthcare sector predicted increased individual adherence to preventive measures.

Barbeito et al. aimed to estimate the association between the level of restriction in different activity fields and the transmission of SARS-CoV-2 in Spain from 15th September 2020 to 9th May 2021. A daily stringency index (ranging from 0 to 1) was created for each of the 50 Spanish provinces. Overall, they found that increasing restrictions by one standard deviation led to a 22% reduction in SARS-CoV-2 transmission within 1 week. They concluded that the highest effects were mainly in the culture and leisure sectors, social distancing measures, indoor restaurants and indoor sports.

Bonney and Grant examined the COVID-19 prevention activities implemented by local health departments (LHDs) in workplaces across the United States. Their survey approach gathered sample information on various aspects, including worker complaints, surveillance, investigations, interactions with employers and businesses, and the capacity of LHDs. Among the LHD respondents, 94% reported investigating COVID-19 cases linked to workplaces. However, nearly half (47%) expressed that they lacked the necessary capacity to effectively handle COVID-19-related workplace safety complaints. The study also found that proactive outreach to prevent COVID-19 spread in workplaces was more likely to occur when LHDs had prior relationships with jurisdiction employers and when their personnel had formal occupational health and safety (OHS) training. Moreover, the size of the LHD predicted the presence of OHS personnel and sufficient financial resources to support workplace investigation and mitigation activities.

Bornand et al. conducted a qualitative study to explore the perceptions, representations, and practices of mask-wearing among the general population during the COVID-19 pandemic in France's Pays de la Loire region. Using semi-structured walking interviews, the researchers surveyed 116 participants in 11 diverse cities. They found that masks represented a shift from ordinary life to pandemic reality but were often perceived as hindrances to breathing, communication, and social interactions. Medical benefits were overlooked, and mask-wearing decisions were influenced by social relationships and vulnerability. Participants' familiarity with mask-wearing varied, with some believing it could not be taught but learned through experience.

The ecological study of Nguyen et al. investigated the COVID-19 average weekly infection fatality rate (AWIFR) during the Delta and Omicron variant periods in 110 countries. The analysis revealed that higher government effectiveness index and a greater proportion of fully vaccinated individuals were associated with lower AWIFR during the Delta period. However, a higher burden of cardiovascular diseases correlated with increased AWIFR. In the Omicron period, AWIFR was positively linked to years lived with disability caused by metabolism disorders and the proportion of the population aged over 65 years. Conversely, a higher proportion of the population vaccinated with booster doses was connected to a better AWIFR outcome. Moreover, an increase in the government effectiveness index was associated with a decrease in AWIFR over both variant periods. On the other hand, higher death rates caused by diabetes and kidney diseases, as well as a larger percentage of the population aged over 65 years, were linked to a significant increase in AWIFR. These findings offer valuable insights for policymakers to tailor strategies in combating the pandemic's impact on different populations.

Kuo and Wen compared the impacts of various factors on the stringency of governmental containment measures through cross-continental comparisons. They measured domestic threat using a country's weekly confirmed case and death numbers, while imported risk was assessed based on a combination of weekly new cases in each country and air passenger traffic between countries. The findings revealed that domestic case numbers were the primary consideration for governments when deciding to increase policy stringency. The study showed that more developed countries tended to implement stricter policies, because they could more likely manage the negative impacts. Moreover, an interaction between case numbers and development level was observed, with countries at the second or third highest development level focusing more on domestic outbreaks than imported risks, while those at the highest level had similar concerns for both.

In conclusion, there is need to address the challenge of finding a balance between implementing NPIs to control the spread of COVID-19 and promoting socio-economic recovery, considering the limitations of vaccine efficacy against emerging variants. The findings of studies in this collection indicate that there are temporal lagged effects and granger causality relationships among various transmission and human mobility variables. Notably, the studies in this collection emphasize the importance of tailor-made strategies for different states and phases of epidemiological transmission. The findings and frameworks can serve as powerful tools for policymakers to design more refined and effective COVID-19 strategies to contribute to better NPIs practices during pandemic responses.

## Author contributions

SA: Conceptualization, Investigation, Supervision, Validation, Writing—original draft, Writing—review and editing. RG: Conceptualization, Investigation, Supervision, Validation, Writing—original draft, Writing—review and editing. OM: Conceptualization, Investigation, Validation, Writing—original draft, Writing—review and editing, Supervision, Visualization. RW: Writing—original draft, Writing—review and editing, Conceptualization, Validation, Visualization.

